# Treatment of Leucocythœmia

**Published:** 1880-06

**Authors:** Alfred Carpenter


### TREATMENT OF LEUCOCYTHŒMIA.

BY ALFRED CARPENTER, M. D.

The author pointed out that there was something wanting in the present plan of dealing with therapeutics, inasmuch as members of the medical profession are continually trying processes for the cure of diseases which have been shown to be useless, and that text-books continue to recommend medicines which have never done any good. He then gave the history in general terms of two cases of leucocythæmia which he had met with in private practice, and in which there were singular symptoms, one being associated with intense neuralgia, the other with recurring priapism. The neuralgia case was treated by means of iron, stimulants, and narcotics. In the opinion of the author, the remedies only increased the intensity of the pain. He always found that the internal as well as the cutaneous administration of narcotics left the patient more sensitive after the effect of the dose had disappeared, that they were useless in arresting the course of the disease. He entered a protest against the cutaneous administration of narcotics as only another form of intoxication, and he objected to medical men making themselves parties to so reprehensible a practice. He had found iron and stimulants unable to arrest the course of leucocythæmia, and he urged that their use was only a waste of time. The second case was treated by means of bromide of potassium, iron, quinine, and turpentine. The priapism had recurred at regular intervals for some time; it had not been controlled by any of the ordinary remedies used, but it seemed to be mastered by the use of galvanism. He deduced five points as worthy of record, and which the author considered to be in a great measure proved by the results of this case (fortified as they were by his experience in the treatment of others). Point 1 was that bromide of potassium did not arrest the course of the disease, and had no effect upon the enlargement of the spleen in this disease. Point 2, that quinine did not have any beneficial effect in leucocythæmia, and it seemed by this result to separate the disease entirely from those affections of the spleen which are associated with malaria; that even in the large doses which were given for nineteen days, viz., twenty-grain doses three times daily—there was no reduction of temperature, and no decided alteration in the course which the disease took, the daily rise of temperature being the same as before quinine was administered. The 3rd point, that the hæmorrhagic tendency (which is one of the symptoms of the disease) was not in any way arrested by the use of perchloride of iron. Point 4 was shown in the inability of turpentine to stay its progress, and which seemed to show that iron and turpentine would be beneficial in those cases in which the blood had not altered from its natural state, but that both were useless in conditions such as arose in leucocythæmia. The 5th point was that aperients were worse than useless. The author concluded by pointing out the possible connexion between the disease and eczema. All the cases he had met with had been preceded by that disease, and he asked members of the Society to give a good trial to arsenic in any cases which might come to their notice, and to publish the result, but not to waste their energies in using bromides, quinine, salicin, iron, turpentine, stimulants, or narcotics.—*London Lancet.*

